# Assessing Hemodialysis Catheter Insertion Practices Against CDC Guidelines: A Closed-Loop Audit at a Tertiary Hospital in Egypt

**DOI:** 10.7759/cureus.71319

**Published:** 2024-10-12

**Authors:** Youssef A Ahmed, Habiba E Hussen, Ahmed M Elbarawy, Osama R Hamza, Hesham A Elghoneimy

**Affiliations:** 1 Internal Medicine Department, Nephrology Unit, Faculty of Medicine, Alexandria University, Alexandria, EGY

**Keywords:** catheter-related infections, centers for disease control and prevention, guideline adherence, hemodialysis unit, renal dialysis

## Abstract

Background

Following evidence-based guidelines during hemodialysis catheter insertion procedures is key to lowering catheter-related bloodstream infections (CRBSI) and other complications. Our aim was to examine all aspects relevant to the insertion of hemodialysis catheters in the dialysis unit at Alexandria Main University Hospital, Alexandria, Egypt, in order to identify practices that could be contributing to high complication rates.

Methods

We used the CDC’s Guidelines for the Prevention of Intravascular Catheter-Related Infections to define the criteria relevant to hemodialysis catheter insertion procedures. These criteria were made into a form to be used as the primary auditing tool. The form was then used to record catheterization procedures in the dialysis unit from March 2024 to July 2024. Eighty patients scheduled for hemodialysis catheter insertion were randomly selected, 40 in the initial cycle of the audit, and 40 in the re-audit cycle. Percentage adherence to each criterion was then calculated for each audit cycle and compared.

Results

Overall, adherence to the criteria assessed showed significant improvements, with mean compliance increasing from 69% in the initial cycle to 90% in the second cycle. In addition, the rate of mechanical complications recorded decreased.

Conclusion

Our study emphasized the effectiveness of using closed-loop clinical audits to ensure continuous improvement of care delivered during hemodialysis catheter insertion procedures.

## Introduction

While hemodialysis catheters are lifesaving for patients with acute kidney injury and chronic kidney disease, if done without following the proper guidelines, they could lead to devastating complications, one of which is catheter-related bloodstream infections (CRBSI). Many of these complications, including CRBSI, can be prevented by complying with evidence-based guidelines. 

CRBSI is a major cause of morbidity and mortality among hemodialysis patients [1]. The implementation of evidence-based guidelines is paramount to lowering the occurrence of CRBSI [2]. Rates of mechanical complications, such as inadvertent arterial punctures and hematoma formation, can also be reduced significantly by following proper procedures. 

A clinical audit is a quality improvement strategy aimed at improving clinical practice by systematically examining processes against guidelines of best practice. This helps identify areas where improvement is needed to provide optimal patient care. It is highly encouraged by the National Institute of Clinical Excellence (NICE) to improve patient outcomes [3,4]. 

Following evidence-based guidelines is the key to providing optimal care and preventing unnecessary complications. It has been demonstrated that clinical audits have a role in encouraging adherence to guidelines related to central venous catheters (CVCs) by quantifying percentage adherence [5-7]. 

The Centers for Disease Control and Prevention (CDC) has provided comprehensive guidelines pertaining to the prevention of intravascular catheter-related infection [2]. Since our institution relies on the CDC when it comes to infection control measures, we decided to apply their evidence-based guidelines to define the criteria evaluated during this clinical audit. The guidelines relevant to the insertion of central hemodialysis catheters were extracted into a checklist, which was used as the main auditing tool. 

Our study aims to evaluate whether catheter insertion practices at the dialysis unit in Alexandria Main University Hospital, Alexandria, Egypt, comply with ideal clinical practice standards. In addition, the study aims to assess whether auditing is a feasible method of implementing changes necessary for improving hemodialysis catheter insertion procedures in the dialysis unit at our hospital. Based on a comprehensive literature review, this is the first prospective audit on hemodialysis catheter insertion practices from Egypt, using the CDC’s guidelines for the prevention of intravascular catheter-related infections.

## Materials and methods

This prospective closed-loop clinical audit was conducted at the dialysis unit in Alexandria Main University Hospital over the span of five months, from March 2024 to July 2024. Eighty patients scheduled for hemodialysis catheter insertion were randomly selected, 40 in the initial cycle of the audit, and 40 in the re-audit cycle. 

Data collection 

Non-tunneled and tunneled hemodialysis catheter insertions were observed by junior doctors in the procedure room of the dialysis unit. A checklist was filled out for each case. The checklist was made by extracting the guidelines relevant to hemodialysis catheter insertion procedures from the CDC’s bloodstream infection guidelines [2]. The criteria assessed are shown below in Table 1. Furthermore, possible mechanical complications [8], including hematomas, inadvertent arterial punctures, hemothorax, and pneumothorax were added to the checklist, to gather data on their incidence. 

**Table 1:** Criteria used to assess hemodialysis catheter insertion procedures extracted from CDC guidelines CDC Guidelines for the Prevention of Intravascular Catheter-Related Infections [2]

Criteria	Response	
Avoid the subclavian catheter insertion in hemodialysis patients and patients with advanced kidney disease	Yes	No
If chronic renal failure patient, catheter used temporarily until fistula or graft ready for permanent access	Yes	No
Perform hand hygiene before insertion	Yes	No
Aseptic technique maintained during insertion	Yes	No
Sterile gloves worn for insertion of the catheter	Yes	No
Use maximal sterile barrier precautions, including the use of a cap, mask, sterile gown, sterile gloves, and a sterile full-body drape	Yes	No
Prepare clean skin with a >0.5% chlorhexidine preparation with alcohol before catheter insertion (in case of chlorhexidine being contraindicated, use a tincture of iodine, an iodophor, or 70% alcohol as an alternative)	Yes	No
Use ultrasound guidance to place catheters to reduce the number of cannulation attempts and mechanical complications (if guidewire exchange is being performed, the use of ultrasound guidance is not applicable)	Yes	No
Use povidone iodine antiseptic ointment or bacitracin/gramicidin/polymyxin B ointment at the hemodialysis catheter exit site after catheter insertion	Yes	No
Use either sterile gauze or sterile, transparent, semipermeable dressing to cover the catheter site	Yes	No
Do not administer systemic antimicrobial prophylaxis routinely before insertion or during the use of an intravascular catheter to prevent catheter colonization or catheter-related bloodstream infections (CRBSI)	Yes	No
Encourage patients to report any changes in their catheter site or any new discomfort to their provider	Yes	No

Interventions 

Multiple methods were utilized in an attempt to improve adherence to the guidelines based on the results of the first audit cycle. Firstly, a seminar took place after the first audit cycle was completed to inform nephrology residents of the results, and recommendations were made to overcome pitfalls in each criterion. 

**Figure 1:**
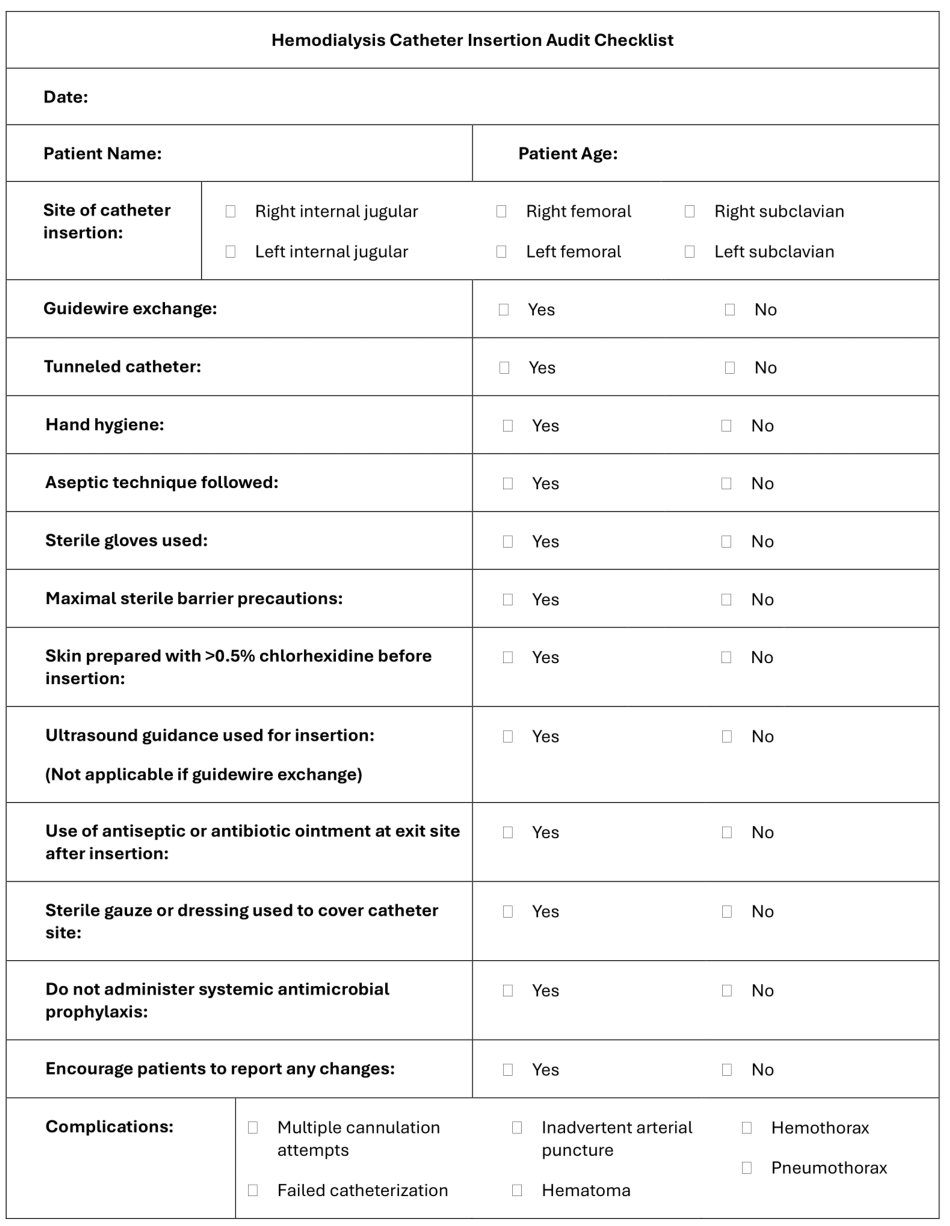
Hemodialysis catheter insertion procedure checklist Adapted from [2]

A criteria checklist was provided to be used by junior residents and doctors in the procedure room to ensure all applicable criteria were fulfilled in each case (Figure 1). 

A workshop was also held for the dialysis unit staff, including the nursing team, to address the shortcomings in the initial audit cycle regarding catheter care immediately after catheter placement. This was also necessary to ensure the dialysis staff were up-to-date with guidelines relevant to hemodialysis catheters.

**Figure 2:**
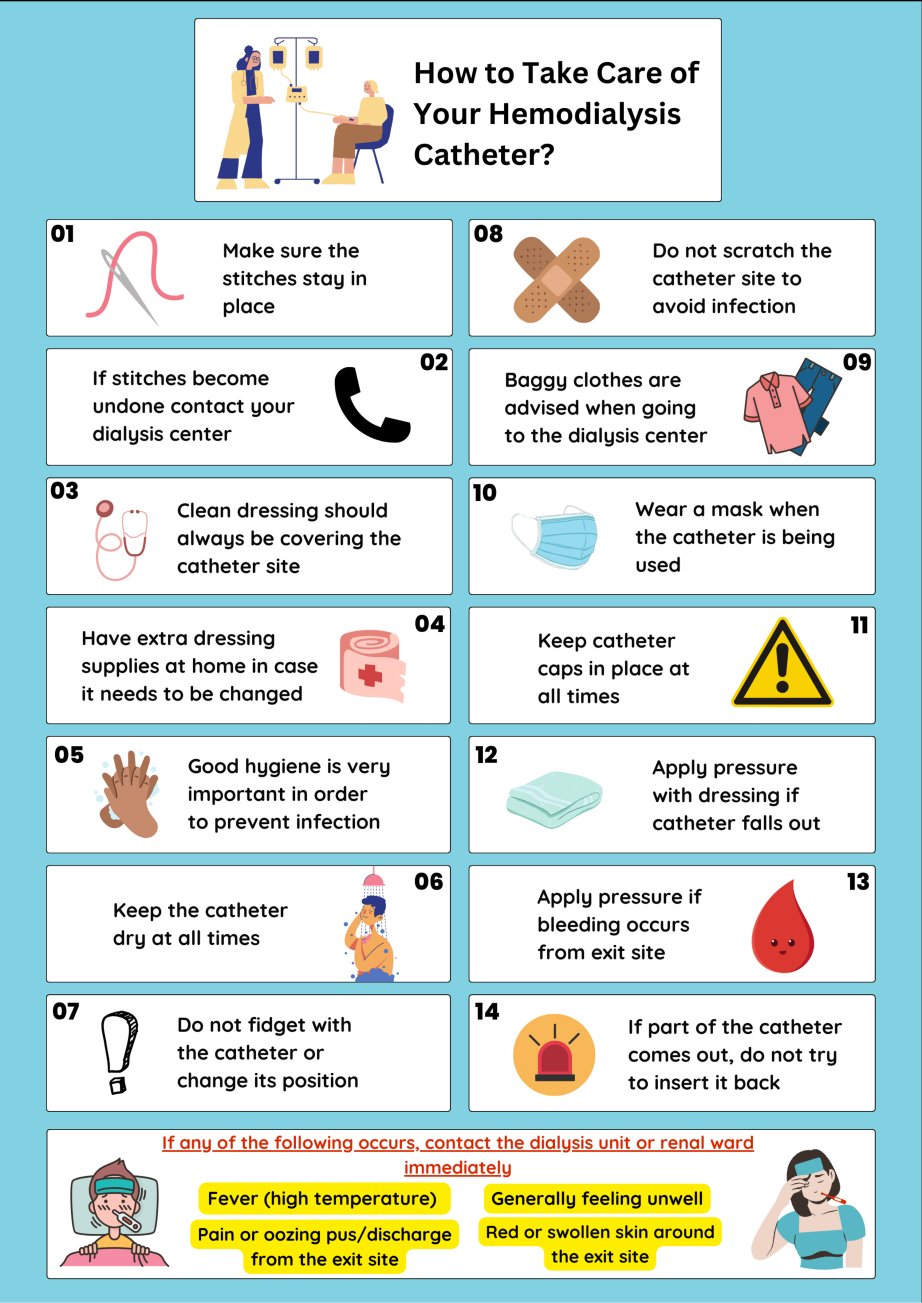
Infographic aimed at hemodialysis patients Adapted from [9].

In addition, doctors were encouraged to engage more in patient education regarding hemodialysis catheters. Considering patients generally respond well to visual aids, crucial points regarding catheter care were made into an infographic [9-11]. The infographic is shown in Figure 2. Since Arabic is the language primarily spoken in Egypt, it was also translated and displayed in Arabic. The infographic was also printed in the form of pamphlets and given to patients upon discharge, which motivated patients to be proactive in their catheters' care. Moreover, a seminar was held for nephrology residents focusing on important information that should be made clear to hemodialysis patients before they are discharged.

Data analysis 

The data collected was used to calculate percentage adherence to each criterion and mean compliance to criteria in each cycle of the audit using IBM SPSS Statistics for Windows, Version 27, (Released 2020; IBM Corp., Armonk, New York, United States). The incidence of recorded complications was also calculated as a percentage of the total cases in each cycle. Descriptive analysis was used to compare the data obtained from both audit cycles. The study protocol was accepted by the Alexandria University Ethics Committee. The procedures adhered to the guidelines of the Declaration of Helsinki (1996). 

## Results

Table 2 shows the percentage adherence to the criteria assessed during both cycles of the audit.

**Table 2:** Comparison of adherence to each criterion between the initial audit cycle and the re-audit cycle

Criteria	Initial audit	Re-audit	% increase in adherence
Avoid the subclavian catheter insertion in hemodialysis patients and patients with advanced kidney disease	100%	100%	-
If chronic renal failure patient, catheter used temporarily until fistula or graft ready for permanent access	100%	100%	-
Perform hand hygiene before insertion	98%	98%	-
Aseptic technique maintained during insertion	78%	98%	20%
Sterile gloves worn for insertion of the catheter	100%	100%	-
Use maximal sterile barrier precautions, including the use of a cap, mask, sterile gown, sterile gloves, and a sterile full-body drape	35%	85%	50%
Prepare clean skin with a >0.5% chlorhexidine preparation with alcohol before catheter insertion (In case of chlorhexidine being contraindicated use a tincture of iodine, an iodophor, or 70% alcohol as alternatives)	58%	63%	5%
Use ultrasound guidance to place catheters to reduce the number of cannulation attempts and mechanical complications (if guidewire exchange is being performed, the use of ultrasound guidance is not applicable)	21%	72%	51%
Use povidone iodine antiseptic ointment or bacitracin/gramicidin/polymyxin B ointment at the hemodialysis catheter exit site after catheter insertion	43%	95%	53%
Use either sterile gauze or sterile, transparent, semipermeable dressing to cover the catheter site	85%	100%	15%
Do not administer systemic antimicrobial prophylaxis routinely before insertion or during use of an intravascular catheter to prevent catheter colonization or CRBSI	100%	100%	-
Encourage patients to report any changes in their catheter site or any new discomfort to their provider	10%	70%	60%
Mean adherence	69%	90%	21%

In general, there was a significant improvement in adherence to the guidelines. Mean percentage adherence increased from 69% to 90%. Some guidelines, including avoiding subclavian catheters in hemodialysis patients, prioritizing fistulas/grafts in case of chronic dialysis patients, performing hand hygiene, using sterile gloves, and not administering prophylactic antibiotics, were almost ideally followed from the start. 

**Figure 3:**
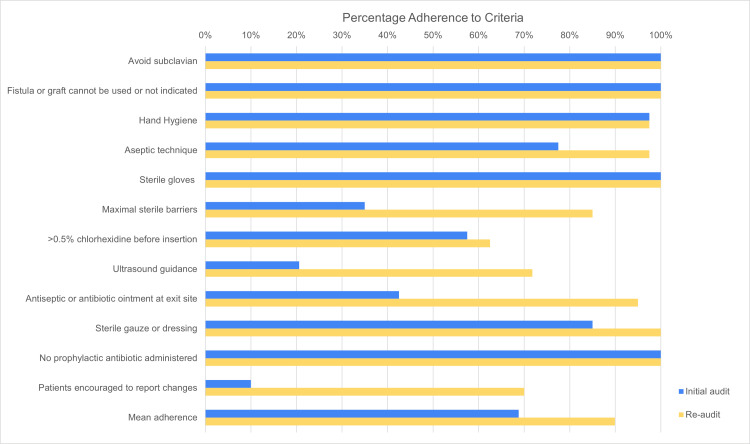
Percentage adherence to each criterion in both cycles of the audit

The criterion showing the smallest improvement was the use of >0.5% chlorhexidine preparation with alcohol to prepare the skin before the procedure. Meanwhile, the criterion involving patient education showed the greatest improvement, followed by the use of antiseptic/antibiotic ointment at the exit site, the use of ultrasound guidance, and the use of maximal sterile barrier precautions. Figure 3 shows a summary of the data. 

**Figure 4:**
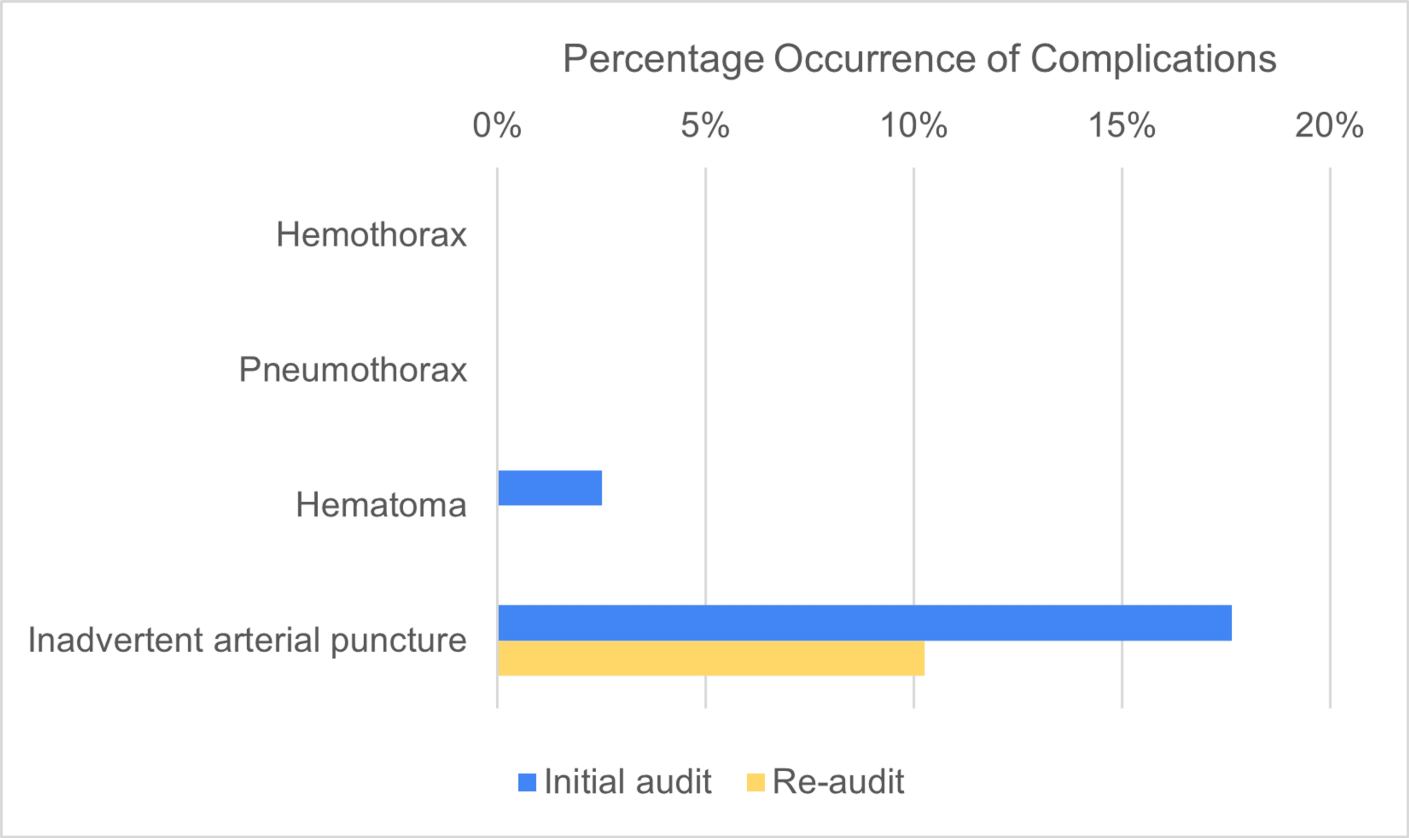
Chart comparing the incidence of complications in both cycles of the audit

The incidence of mechanical complications was also recorded. As shown in Table 3 and Figure 4, the incidence of iatrogenic pneumothorax and hemothorax was 0% in both cycles of the audit. While hematomas and inadvertent arterial punctures occurred in some cases, their incidence decreased in the second cycle of the audit compared to the first cycle. 

**Table 3:** Incidence of complications in both cycles of the audit

Complication	Initial audit	Re-audit
Hemothorax	0%	0%
Pneumothorax	0%	0%
Hematoma	3%	0%
Inadvertent arterial puncture	18%	10%

## Discussion

**Figure 5:**
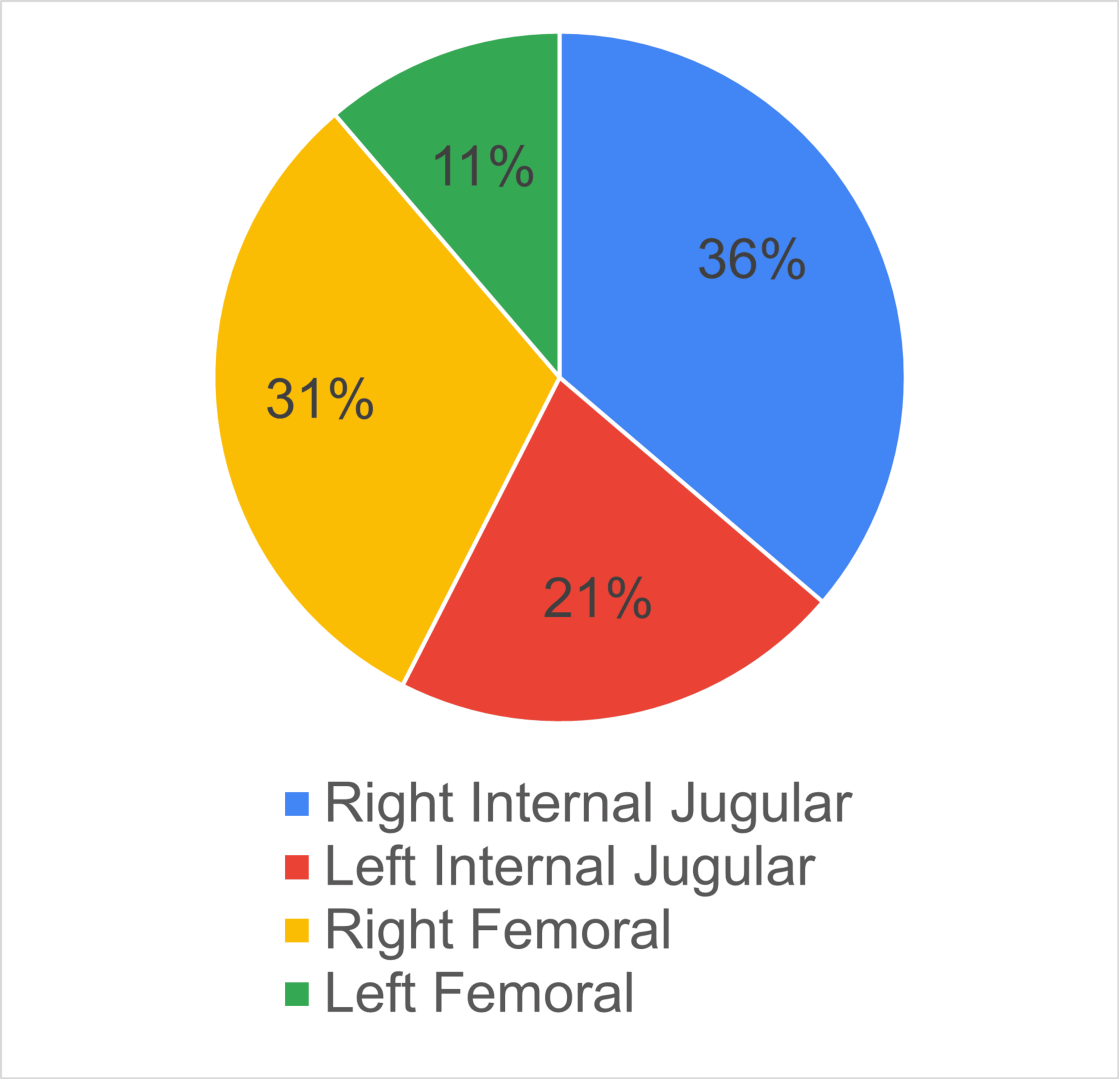
Chart demonstrating the access sites used for hemodialysis catheters during the audit

As shown in Figure 5, no subclavian catheter insertions were performed, which is in accordance with the guideline that mentions avoiding the subclavian site for hemodialysis catheters. However, considering that 42% of catheters recorded were in the femoral site, in future audits, the indication of femoral catheters should be closely examined to ensure they are not used unnecessarily, since femoral catheters increase the risk of deep vein thrombosis as well as infection in obese patients [12,13]. 

At the dialysis unit where the audit took place, fistulas/grafts were prioritized as access for dialysis in chronic renal patients. Hemodialysis catheters were only inserted in acute indications of dialysis, in chronic patients preparing for fistula/graft surgeries, and in cases where arteriovenous fistula/graft access was contraindicated [14]. Furthermore, adherence to hand hygiene, use of sterile gloves, and not administering systemic antibiotics as prophylaxis in the absence of infection were nearly ideal during both cycles of the audit. 

The criterion that showed the least improvement was the use of >0.5% chlorhexidine antiseptic to prepare the insertion site. During the seminars, it was clear that many doctors and nurses thought povidone-iodine and chlorhexidine preparation were equally effective as an antiseptic. Even though most nephrology residents were made aware that chlorhexidine solution is superior to povidone-iodine as an antiseptic [2], compliance with this guideline showed only a modest increase. When the re-audit results were discussed in the department, it was apparent that this was because there was a shortage of chlorhexidine preparation in the hospital. However, when given the choice, doctors and nurses now use >0.5% chlorhexidine preparation with alcohol instead of povidone-iodine. 

The lower incidence of mechanical complications, namely hematoma formation and inadvertent arterial punctures, during the second cycle of the audit, is likely linked to the 51% increase (from 21% to 72%) in the use of ultrasound guidance for placement of the catheters. Adherence to ultrasound guidance however was still lacking, due to only one ultrasound machine being readily available for use. 

The aseptic technique was almost perfectly adhered to in the second cycle of the audit compared to the initial cycle. Notably, workshops held for the nursing team of the dialysis unit led to significant improvements in immediate catheter exit site care after insertion. The use of both antiseptic/antibiotic ointment and sterile gauze/sterile dressing at the exit site notably increased. 

The last criterion assessed which was related to patient education showed a significant increase in adherence during the second cycle compared to the baseline audit. The low compliance to this domain was mostly due to doctors mainly relying on nurses of the dialysis unit for patient education. Residents were encouraged to take advantage of most patient encounters for patient communication and inquiries regarding catheter care. It was also found that patients responded well to pamphlets and posters and were motivated to be involved in their own catheters' care and longevity. The infographic (Figure 2) also served to remind doctors and nurses in the dialysis unit of points [9] that should be known by patients before discharge. 

Recommendations to improve standard practice 

Recommendations and updates found in the CDC’s guidelines were presented in the department to discuss updating the standards of practice for managing hemodialysis catheters. This includes the use of chlorhexidine-impregnated dressing, lock solution antibiotic in long-term catheters in patients with a history of multiple episodes of CRBSI, and the use of two sets of sterile gloves during guidewire exchanges [2]. 

Encouraging junior doctors to have a systematic approach to standard practice using simple checklists before and after the procedure will contribute to improved overall adherence to guidelines and a reduction in the rates of complications. 

With junior doctors and medical interns becoming keener on clinical auditing at the hospital, workshops could be held on systematic auditing of catheter placement procedures to encourage regular auditing at the dialysis unit. Making sure healthcare personnel including doctors and nurses are up to date on new recommendations between audit cycles is also essential. 

There are other aspects of catheter care, such as scrub-the-hub practices [15] and day-to-day catheter dressing regimens that need standardized audit programs to make sure all aspects of catheter care and handling are optimal. 

## Conclusions

Auditing is an excellent way of maintaining high-quality clinical practice and keeps healthcare personnel striving to improve patient care. This study highlighted the importance of systematic auditing in maintaining satisfactory clinical practice and encouraging regular auditing in hemodialysis units where regular catheter insertions take place. In addition, it was proposed that rates of CRBSI could be measured in future clinical audits to identify trends in CRBSI rates over time. This will improve adherence to infection control guidelines, which will reduce CRBSI rates and other complications, ultimately lowering patient morbidity and mortality.
